# Impaired Glucose-Insulin Metabolism in Multisystem Inflammatory Syndrome Related to SARS-CoV-2 in Children

**DOI:** 10.3390/children8050384

**Published:** 2021-05-13

**Authors:** Valeria Calcaterra, Pietro Bosoni, Dario Dilillo, Savina Mannarino, Laura Fiori, Valentina Fabiano, Patrizia Carlucci, Elisabetta Di Profio, Elvira Verduci, Chiara Mameli, Gloria Pelizzo, Elena Zoia, Lucia Sacchi, Cristiana Larizza, Gianvincenzo Zuccotti

**Affiliations:** 1 Pediatric and Adolescent Unit, Department of Internal Medicine, University of Pavia, 27100 Pavia, Italy; 2 Pediatric Department, “Vittore Buzzi” Children’s Hospital, 20154 Milan, Italy; dario.dilillo@asst-fbf-sacco.it (D.D.); laura.fiori@asst-fbf-sacco.it (L.F.); valentina.fabiano@unimi.it (V.F.); patrizia.carlucci@asst-fbf-sacco.it (P.C.); elisabetta.diprofio@unimi.it (E.D.P.); elvira.verduci@unimi.it (E.V.); chiara.mameli@unimi.it (C.M.); gianvincenzo.zuccotti@unimi.it (G.Z.); 3 Department of Electrical, Computer and Biomedical Engineering, University of Pavia, 27100 Pavia, Italy; pietro.bosoni02@universitadipavia.it (P.B.); lucia.sacchi@unipv.it (L.S.); cristiana.larizza@unipv.it (C.L.); 4 Pediatric Cardiology Unit, Pediatric Department, “Vittore Buzzi” Children’s Hospital, 20154 Milano, Italy; savina.mannarino@asst-fbf-sacco.it; 5 Department of Biomedical and Clinical Science “L. Sacco”, University of Milan, 20157 Milan, Italy; gloria.pelizzo@gmail.com; 6 Department of Health Sciences, University of Milano, 20142 Milano, Italy; 7 Pediatric Surgery Department, “Vittore Buzzi” Children’s Hospital, University of Milan, 20154 Milan, Italy; 8 Anesthesia and Intensive Care Unit, Pediatric Department, “Vittore Buzzi” Children’s Hospital, 20154 Milano, Italy; elena.zoia@asst-fbf-sacco.it

**Keywords:** multisystem inflammatory syndrome, SARS-CoV-2, glucose, insulin, children

## Abstract

An interaction between metabolic glucose impairment and coronavirus disease 2019 is reported. The development of a severe multisystem inflammatory syndrome in children (MIS-C) related to SARS-CoV-2 infection has been described. We evaluated the impact of MIS-C on glycemic patterns in pediatric patients. A group of 30 children and adolescents affected by MIS-C were considered; all patients were normal weight. Clinical and biochemical assessments, including surrogate markers of insulin resistance (IR) such as homeostasis model analysis-IR (HOMA-IR) and triglyceride–glucose (TyG) indexes, were recorded. Patients were also invited to undergo an intermittently scanned continuous glucose monitoring (isCGM). HOMA-IR index was calculated in 18 patients (60%), of which 17 (94%) revealed a pathological value. TyG index was computed for all patients and pathological values were detected in all cases. In 15 patients, isCGM data were recorded on average for 9 days (±3 days). Overall, average glucose was 105 mg/dL (±16 mg/dL) and average time spent in the 70–180 mg/dL range (TIR) was 93.76%, with nearly 10% of glucose readings in the 141–180 mg/dL range; glycemic fluctuations over the hyperglycemic threshold were detected in four patients. Regular glucose monitoring may be useful to prevent metabolic imbalance and obtain a better outcome.

## 1. Introduction

The literature supports an active interaction between metabolic glucose impairment and coronavirus disease 2019 (COVID-19) [1]. As reported by Hoffmann, hyperglycemia and glycemic fluctuations may be caused by the inflammatory cascade associated with the attack of severe acute respiratory syndrome coronavirus 2 (SARS-CoV-2) on the pancreas and the potentially impaired β-cell function [2].

During the pandemic children have been less affected than adults and SARS-CoV-2 was in most cases asymptomatic or with mild symptoms [3,4,5]. However, the development of a severe multisystem inflammatory syndrome in children (MIS-C) related to SARS-CoV-2 infection has been reported [6,7,8,9]. As stated by the Center of Diseases Control and Prevention (CDC), the definition of MIS-C requires patients to be less than 21 years and to have evidence of either recent/current SARS-CoV-2 infection or exposure within the 4 weeks prior to the onset of symptoms, the presence of documented fever, elevated markers of inflammation, at least two signs of multisystem involvement, and finally, lack of an alternative diagnosis (e.g., bacterial sepsis, toxic shock syndrome) [10].

Hyperglycemia is a common complication in critically ill non-diabetic children and represents a stress response due to peripheral insulin resistance (IR), relative insulin deficiency, and glucose metabolism impairment [11,12]; the effects of medicaments such as catecholamine, glucocorticoids, and exogenous dextrose administration may also be considered [12]. Although tight glucose control is not related to a significant reduction in hospital mortality [13], blood glucose variability is associated with multiorgan dysfunction [14], and longer duration of hyperglycemia is an independent factor related to mortality [15].

Even though MIS-C represents a critical health condition associated with SARS-CoV-2 infection [6,7,8,9], data evaluating glucose disorders have not previously described. This exploratory study was conducted to evaluate the impact of MIS-C on glycemic patterns in pediatric patients admitted to Vittore Buzzi Children’s Hospital in Milan, Italy, during the pandemic.

## 2. Materials and Methods

### 2.1. Subjects

We recruited a group of 30 Italian children and adolescents admitted between 1 November 2020 and 9 January 2021 to the Pediatric Department of Vittore Buzzi Children’s Hospital (Milan, Italy) for MIS-C, defined according to the CDC classification [10]. Children with a known history of diabetes mellitus and/or insulin resistance, assumption of steroid/drug inducing hyperglycemia at admission, and suspected or proven inborn errors of metabolism were excluded.

For all patients, a clinical and biochemical assessment was recorded on admission. Moreover, patients were invited to undergo intermittently scanned continuous glucose monitoring (isCGM) through the FreeStyle Libre flash glucose monitoring (FGM) system (Abbott Diabetes Care, Alameda, CA, USA) [16]. The isCGM sensor was applied by caregivers at the back of patients’ upper arm before the start of MIS-C therapy.

The study was conducted according to the guidelines of the Declaration of Helsinki and approved by the Institutional Review Board of the hospital (protocol number 2021/ST/004). Children’s guardians gave their written consent for inclusion after being informed about the nature of the study.

### 2.2. Measurements and Statistical Analysis

Physical examination included anthropometric measurements of weight and height, and evaluation of the pubertal stage [17,18]. Body Mass Index (BMI) was calculated by dividing the patient’s weight in kilograms by the square of the height in meters and then transformed into BMI z-scores using the WHO reference values [19]. The diagnostic procedure for confirming the MIS-C diagnosis included a complete blood count and measurements of C-reactive protein (CRP), procalcitonin, ferritin, cardiac troponin T (cTnT), N-Terminal pro-Brain Natriuretic Peptide (NT-proBNP), coagulative parameters, creatine kinase, electrolytes, and interleukin-6 (IL-6). The therapeutic protocol involved intravenous immunoglobulin and corticosteroid.

Additionally, at admission, the metabolic profile including total and HDL cholesterol, fasting plasma glucose (FPG), insulin (FPI) and triglycerides (TG) was analyzed (a blood sample was obtained in fasting state between 8:30 a.m. and 9:00 a.m.) [20].

Two indexes were used as a surrogate of insulin resistance (IR):

- Homeostasis model analysis—insulin resistance (HOMA-IR) index, defined as ([fasting plasma insulin (mU/L) × fasting plasma glucose (mg/dL)]/405) [21]; the cutoff point for pathological IR was set at the 97.5th percentile of the HOMA-IR distribution in a representative group of Italian healthy children and adolescents grouped by sex and pubertal stage [22].
- Triglyceride–glucose (TyG) index, calculated as (ln [fasting triglycerides (mg/dL) × fasting plasma glucose (mg/dL)/2]) [23,24]; the cutoff point for pathological IR was set at 7.88 [20,25].

A pairwise qualitative and quantitative analysis was performed between IR indexes and each variable (excluding FPG, FPI, and TG, since they have been used for the computation of HOMA-IR and TyG indexes). After the execution of Shapiro–Wilk’s statistical tests, which revealed that a number of variables were not normally distributed, the non-parametric Spearman ρ was used to estimate the correlation between each IR index and clinical and biochemical parameters [26].

The isCGM data were investigated through the computation of the most useful metrics in clinical practice according to the Advanced Technologies and Treatments for Diabetes (ATTD) consensus recommendations [27], such as:

- Average glucose;
- Glucose standard deviation (SD);
- Time below range (TBR), i.e., the percentage of glucose readings under 70 mg/dL, which can be further divided into time slightly below range in the 54–69 mg/dL range, and time severely below range under 54 mg/dL;
- Time in range (TIR), i.e., the percentage of glucose readings in the 70–180 mg/dL range, which can be further divided into time in the 70–140 mg/dL target range (TIT), and time in the 141–180 mg/dL range;
- Time above range (TAR), i.e., the percentage of glucose readings over 180 mg/dL, which can be further divided into Time slightly above range in the 181–250 mg/dL range, and time severely above range over 250 mg/dL.

All the analyses were performed using the R system for statistical computing, version 4.0.4.

## 3. Results

All 30 patients were normal weight [19]; no one showed acanthosis nigricans. Participants’ clinical and biochemical characteristics on admission are presented in Table 1.

Due to some missing FPI data, it was possible to calculate the HOMA-IR index for only 18 patients (60%). Among these, 17 (94%) revealed a pathological HOMA-IR value. Instead, the TyG index was computed for all patients and pathological values were detected in all cases.

**Figure 1:**
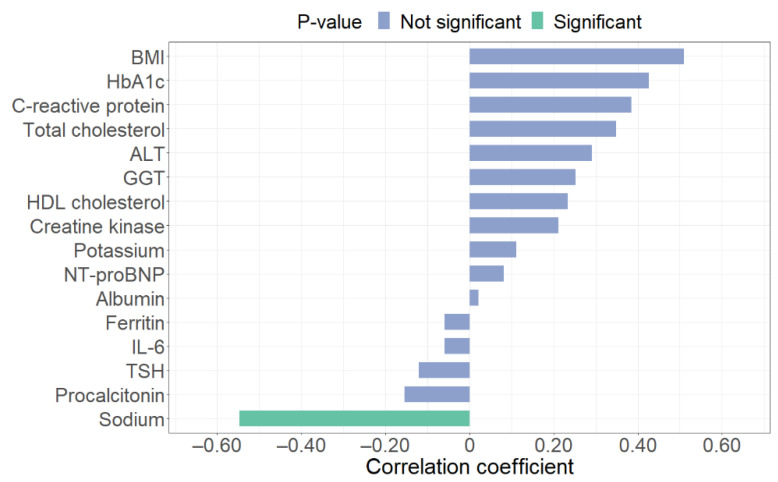
Spearman correlation coefficients between clinical and biochemical parameters and homeostasis model analysis—insulin resistance (HOMA-IR) index. BMI: Body Mass Index; HbA1c: glycated hemoglobin; ALT: alanine transaminase; GGT: gamma-glutamyl transferase; NT-proBNP: N-Terminal pro-Brain Natriuretic Peptide; IL-6: interleukin-6; TSH: thyroid-stimulating hormone.

**Figure 2:**
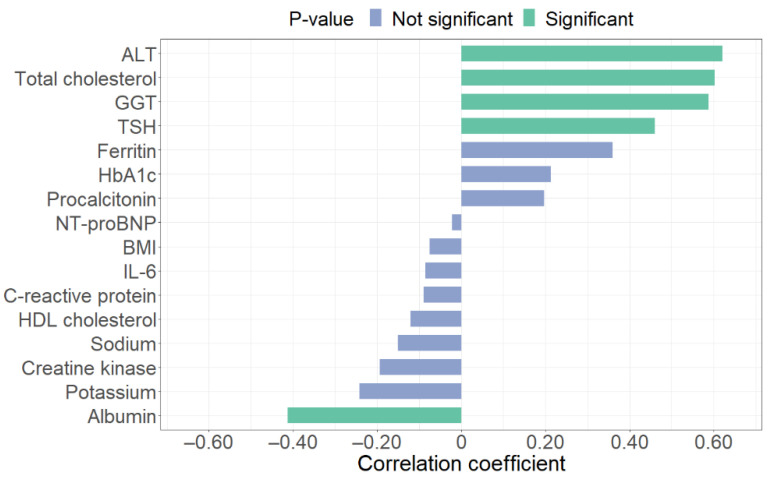
Spearman correlation coefficients between clinical and biochemical parameters and triglyceride–glucose (TyG) index. ALT: alanine transaminase; GGT: gamma-glutamyl transferase; TSH: thyroid-stimulating hormone; HbA1c: glycated hemoglobin; NT-proBNP: N-Terminal pro-Brain Natriuretic Peptide; BMI: Body Mass Index; IL-6: interleukin-6.

Bar plots in Figure 1 and Figure 2 display the correlation coefficients of each IR index, highlighting (in green) the relationships that result statistically significant (*p*-value < 0.05). Respectively, sodium has a significant correlation with HOMA-IR (*p*-value = 0.02), while ALT (*p*-value < 0.01), total cholesterol (*p*-value < 0.01), GGT (*p*-value < 0.01), TSH (*p*-value < 0.01), and albumin (*p*-value = 0.02) revealed a significant correlation with TyG index.

**Figure 3:**
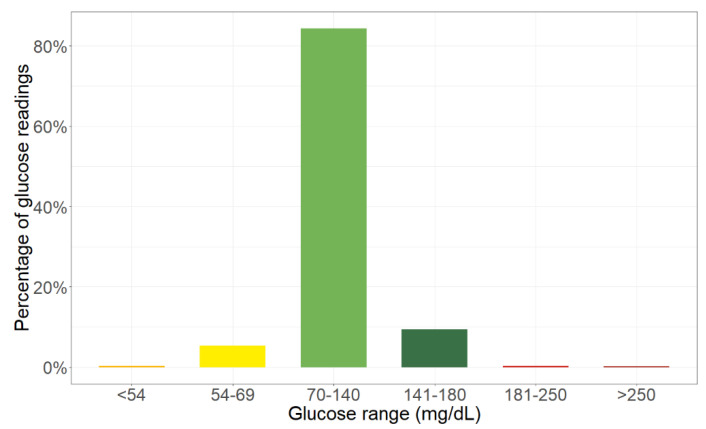
Bar representation of average time in ranges.

Overall, the average glucose value is 105 mg/dL (±16 mg/dL SD). Figure 3 shows the mean percent partition of time spent within different glucose ranges. Average TIR is 93.76% (±9.94% SD) with 9.42% (±14.86% SD) of glucose readings in the 141–180 mg/dL range (dark green bar). Time spent outside the 70–180 mg/dL range is highly asymmetrical: average TBR is 5.67% (±10.19% SD), with only 0.30% (±0.64% SD) of glucose readings (orange bar) under the 54 mg/dL threshold of severe hypoglycemia, while average TAR (red bars) is 0.57% (±1.20% SD).

**Figure 4:**
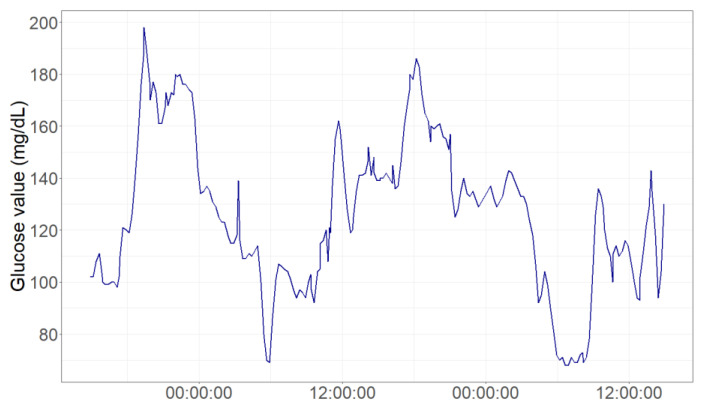
Glycemic fluctuations in a patient 48-h monitoring window.

Specifically, four patients reveal glycemic fluctuations over the 180 mg/dL threshold of hyperglycemia, as shown by the time series chart in Figure 4.

## 4. Discussion

MIS-C is a critical illness in children and adolescents, appearing several weeks after initial infection [6,7,8,9]. Thus far, no universally agreed upon approach is available for this syndrome [9] and, to the best of our knowledge, no one has previously described glucose-insulin metabolic disorders in a pediatric population affected by MIS-C. Analyzing our cohort of 30 pediatric patients affected by MIS-C, we noted that IR, glycemic fluctuations and/or hyperglycemia can occur.

Alterations in glucose metabolism are common in severely ill patients. As described for other critical illnesses [11,12], a hypermetabolic state also exists in this kind of patient and the adaptive response allows vital organs to conserve energy. The response appears to be driven by counter-regulatory hormones and cytokines, which may be important mediators of IR, and result in mild to moderate hyperglycemia that provides fuel for the brain and immune system after stress conditions. As happened to two of our patients, an insulin therapy may be necessary for limiting glycemic metabolic imbalance [28].

In our children, the high prevalence of pathological values in HOMA-IR and TyG indexes supports a both hepatic and peripheral impaired insulin action. The action of counter-regulatory hormones on IR in skeletal muscles might be mediated through an increase in the circulating free fatty acid level, despite hyperinsulinemia [11,12]. The correlation between IR markers and lipids, hepatic parameters, thyroid values, electrolytes, and albumin may support the predominance of catabolic condition and the impairment of glucose homeostasis within the body.

As reported in the literature, an interaction between COVID-19 and glucose-insulin metabolic disorders is postulated in adults [1,29] and not excluded in pediatrics [30,31]. In particular, the relationship between COVID-19 and type 2 diabetes mellitus (T2DM) has been extensively described in adults [1,2,30] and a relationship between SARS-CoV-2 infection and type 1 diabetes mellitus (T1DM) has also been discussed in children [30,31,32,33,34,35,36].

This is possibly the first up-to-date study on the relationship between IR and glycemic fluctuation in normal weight children without glycemic disorders. Usually, IR is noted in children and adolescents who are overweight or have moderate to severe obesity. Our results are not surprising in terms of adaptive metabolic response; nevertheless, in this clinical context, a bidirectional relationship between COVID-19 and glycemic impairment could not be excluded. Pancreatic β cells are permissive to SARS-CoV-2 infection with receptor angiotensin-converting enzyme 2 (ACE2) as its entry [37,38]. As considered by Hoffmann [2], hyperglycemia and glycemic fluctuations could be caused by the inflammatory cascade of the attack of SARS-CoV-2 on the pancreas and the potentially impaired β-cell function.

Even though the optimal therapeutic approach to a child with MIS-C has not been defined yet, most patients have been treated with the standard therapeutic protocols including glucocorticoids and intravenous immunoglobulin; thus, the iatrogenic effect on glycemic fluctuation may also be considered. Nevertheless, the evaluation of the HOMA-IR and TyG indexes has been performed at the admission before the start of therapy, as far as possible due to patients’ emergency care. Therefore, the hypothesis of the iatrogenic mechanism of IR can be reasonably excluded.

Hyperglycemia is not a physiological or benign condition; clinically, hyperglycemia has been linked to increased incidences of sepsis, longer hospital stays and higher mortality [39,40,41]. A continuous glucose monitoring may be useful to detect glycemic fluctuation and to prevent metabolic imbalance.

Several limitations should be addressed in this study. Firstly, the small sample size and a male predominance (only seven subjects were females, 23%) might have limited the analyses. Secondly, we were unable to compare isCGM data of the same patients before and after the SARS-CoV-2 infection to obtain an accurate evaluation of the direct influence of COVID-19 on glycemic levels; however, the normal range for HbA1c can support the exclusion of a pre-existing diabetes. Although the gold standard for IR detection is the hyperinsulinemic-euglycemic clamp technique, we used the validated indirect measurement of IR since the clamp in pediatric subjects is cumbersome, time consuming and technically difficult to perform in routine clinical practice. Finally, no genetic susceptibility to type 1 diabetes and/or serum autoantibodies against β-cell antigens were detected. To confirm and strengthen our results, further multicenter collaborative studies are necessary.

## 5. Conclusions

In this exploratory study, we recorded IR and glycemic fluctuation in pediatric patients with MIS-C. Regular glucose monitoring of both fasting and post-prandial glucose levels may be useful for a better outcome. Moreover, these findings also underscore the concerns for new onset diabetes in the COVID-19 pandemic.

## Figures and Tables

**Table 1:** Study participants’ characteristics at baseline. Summary statistics are presented as frequency (percentage) or median ± interquartile range.

Variable	Summary Statistics
Sex	Female: 7 (23.33%) Male: 23 (76.67%)
Age (years)	10.68 ± 7.25
BMI (Kg/m^2^)	17.70 ± 3.99
BMI z-score	0.03 ± 1.49
HbA1c (%)	5.20 ± 0.20
HbA1c (mmol/mol)	33.00 ± 2.25
FPG (mg/dL)	111.00 ± 31.00
FPI (µU/mL)	21.95 ± 11.50
TG (mg/dL)	190.00 ± 177.25
HOMA-IR index	5.15 ± 5.69
TyG index	9.20 ± 0.73
Total cholesterol (mg/dL)	118.00 ± 72.00
HDL cholesterol (mg/dL)	17.00 ± 21.00
TSH (mIU/L)	2.16 ± 1.81
GGT (IU/L)	26.50 ± 38.75
ALT (IU/L)	31.00 ± 45.50
Creatine kinase (IU/L)	68.00 ± 102.00
Albumin (g/L)	25.50 ± 7.50
Sodium (mEq/L)	132.00 ± 5.00
Potassium (mEq/L)	3.50 ± 0.90
Ferritin (µg/L)	745.00 ± 1259.25
IL-6 (ng/L)	83.00 ± 208.50
C-reactive protein (mg/dL)	236.50 ± 176.00
Procalcitonin (µg/L)	6.2 ± 11.20
NT-proBNP (ng/L)	7554.00 ± 11,143.00

BMI: Body Mass Index; HbA1c: glycated hemoglobin; FPG: fasting plasma glucose; FPI: fasting plasma insulin; TG: fasting triglycerides; HOMA-IR: homeostasis model analysis—insulin resistance index; TyG: triglyceride–glucose index; HDL cholesterol: high-density lipoprotein cholesterol; TSH: thyroid-stimulating hormone; GGT: gamma-glutamyl transferase; ALT: alanine transaminase; IL-6: interleukin-6; NT-proBNP: N-Terminal pro-Brain Natriuretic Peptide.

## Data Availability

The data presented in this study are available on request from the corresponding author. The data are not publicly available due to privacy restrictions.
